# Microbiota, Diet and Acute Leukaemia: Tips and Tricks on Their Possible Connections

**DOI:** 10.3390/nu15194253

**Published:** 2023-10-03

**Authors:** Fabiana Furci, Nicola Cicero, Alessandro Allegra, Sebastiano Gangemi

**Affiliations:** 1 Provincial Healthcare Unit, Section of Allergy, 89900 Vibo Valentia, Italy; fabianafurci@gmail.com; 2 Department of Biomedical, Dental, Morphological and Functional Imaging Sciences, University of Messina, Via Consolare Valeria, 98125 Messina, Italy; 3 Division of Hematology, Department of Human Pathology in Adulthood and Childhood “Gaetano Barresi”, University of Messina, Via Consolare Valeria, 98125 Messina, Italy; aallegra@unime.it; 4 Allergy and Clinical Immunology Unit, Department of Clinical and Experimental Medicine, University of Messina, Via Consolare Valeria, 98125 Messina, Italy; gangemis@unime.it

**Keywords:** leukaemia, microbiota, diet, cancer

## Abstract

Acute leukaemia is probably one of the most recurrent cancers in children and younger adults, with an incidence of acute lymphoblastic leukaemia in 80% of cases and an incidence of acute myeloid leukaemia in 15% of cases. Yet, while incidence is common in children and adolescents, acute leukaemia is a rare disease whose aetiology still requires further analysis. Many studies have investigated the aetiology of acute leukaemia, reporting that the formation of gut microbiota may be modified by the start and development of many diseases. Considering that in patients affected by acute lymphoblastic leukaemia, there is an inherent disequilibrium in the gut microbiota before treatment compared with healthy patients, increasing evidence shows how dysbiosis of the gut microbiota provokes an inflammatory immune response, contributing to the development of cancer. Our analysis suggeststhe key role of gut microbiota in the modulation of the efficacy of leukaemia treatment as well as in the progress of many cancers, such as acute leukaemia. Therefore, in this paper, we present an examination of information found in literature regarding the role of dietary factors and gut microbiota alterations in the development of leukaemia and suggest possible future preventive and therapeutic strategies.

## 1. Introduction

Leukaemia, characterised by the unregulated clonal proliferation of haematopoietic stem cells and, after accidents, is the second leading cause of paediatric-aged mortality, with an increase of 0.9% in incidence every year, whose risk factors are currently a topic of study aimed at possible prevention and future therapeutic strategies [1,2,3]. Albeit the precise cause of the disease is not clear, with the exception of the recognised role of ionizing radiation, benzene exposure, some leukaemogenic gene fusions and translocations and that might induce some childhood cases of acute lymphoblastic leukaemia (ALL) with an in-utero origin, many studies are increasingly considering its pathogenetic, infectious and environmental risk factors [4,5,6,7].

**Figure 1:**
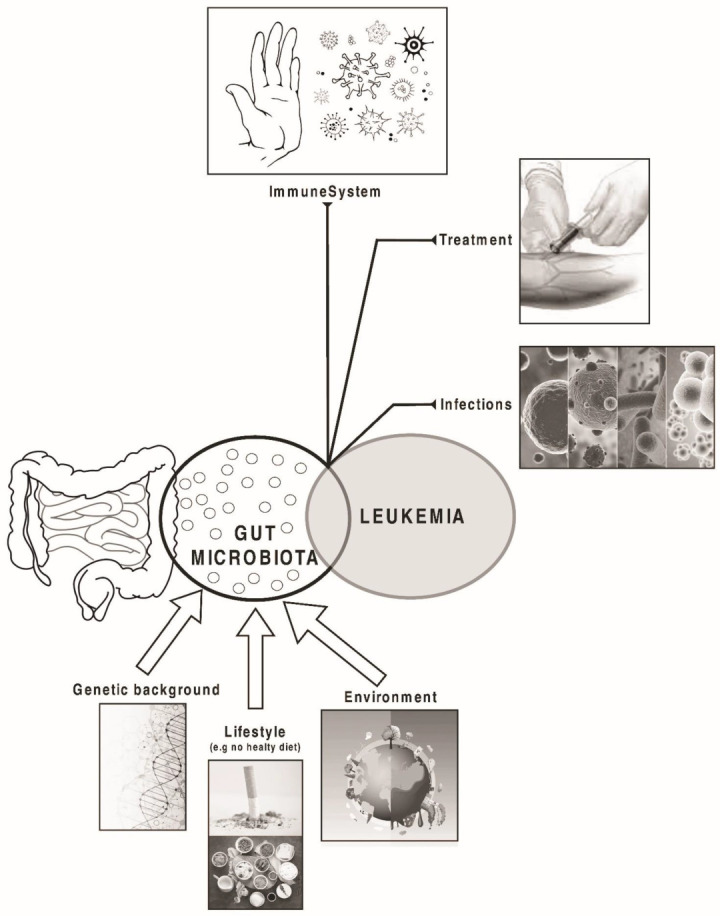
Factors that may influence the composition of gut microbiota and the development and prognosis of leukaemia.

Considering that costs related to leukaemia treatment have a major impact on individual patients and their families, the study of environmental and lifestyle risk factors may impact significantly in the incidence and management of the disease [8] (Figure 1).

Gut microbiota is considered a complex ecosystem, which developed along with the gastrointestinal tract, that can be influenced by many genetic, environmental, and lifestyle factors, along with inherited and acquired genetic disorders [9,10]. In particular, regarding molecular abnormalities that could lead to the deregulation of signalling pathways involved in specific cell behaviour and, therefore, in the development of haematological malignancies, such as the LNK inhibitory adaptor protein encoded by the LNK/SH2B adaptor protein 3 (SH2B3) gene, which is the target of numerous genetic mutations, inherited or acquired in lymphoid leukaemia, high-resolution genome analysis using microarray and large-scale sequencing have both played a key role in the identification of many relevant acquired gene mutations [10].

The role of the microbiota also appears to be closely connected with the response to pharmacological therapies administered to patients with leukaemia, as alterations of the microbiota caused by therapy can influence the prognosis of patients [11,12].

In particular, in this paper, we would like to report our analysis regarding the possible connections among diet, microbiota and acute leukaemia, focusing on the underlying immunological pathways and therefore evaluating possible future therapeutic approaches for the treatment and prevention of leukaemia, raising points for reflection: whether the variability in terms of make-up of gut microbiota might impact the toxicity or efficacy r of drugs administered during the therapeutic management and how; whether allogeneic hematopoietic stem cell transplant (allo-HSCT) or chemotherapy can alter the gut microbiota and how; whether knowledge of gut microbiota can predict the complications due to therapy, such as diarrhoea and infections; and whether the regulation of gut microbiota can help therapy-related complications [1].

Moreover, we also examine the role of the microbiota, whose modulation may improve metabolic health and reduce the risk of cancer, thus indicating potential pathways for precision medicine research in this emerging field.

## 2. The Risk of Leukaemia and Diet: Possible Associations

Various dietary factors play a key role in human health through cellular metabolism, the regulation of gut microbiota and immunological processes [13].

Many studies have reported that the risk of leukaemia is increased in relation to maternal smoking and alcohol consumption or other dietary factors during pregnancy [14,15]. Additionally, the risk of childhood leukaemia can be influenced by maternal diet during pregnancy involving various mechanisms such as the synthesis and repair of DNA and epigenetic factors [16]. Various studies have analysed the link between taking maternal folic acid and the risk of leukaemia in children and have reported that maternal consumption of fruit and vegetables is inversely related to childhood ALL [16,17,18]. In accordance with this result, in California, in a case-control study, evaluating the link between the quality of maternal diet before pregnancy, considering a diet quality index, and risk of childhood ALL and acute myeloid leukaemia (AML), some authors highlighted that a lower risk of childhood ALL may be related with greater maternal consumption of vegetables and fruit and of micronutrients such as folic acid, which act on DNA synthesis and repair or epigenetic processes [19,20].

On the other hand, maternal malnutrition and low levels of micronutrients could cause elevated concentrations of maternal cortisol, influencing foetal immune system development, could interfere with the normal proliferation of immune cells and organogenesis and could modify the quantity and quality of immunological factors which transfer prenatally through the placenta or postnatally through breastfeeding [21,22].

In relation to the risk of ALL, research has also reported a low risk of this disease in relation to a diet that includes fish, seafood, beans and beef [18,23,24]. Instead, the risk of ALL may be increased when mothers eat various foods such as sugars or syrups [25].

As reported in a recent systematic review, there is a possible inverse association between a maternal diet rich in fruit and vegetables during pregnancy and the risk of ALL in children, while there is a positive association between the risk of this disease on drinking more coffee and/or caffeinated beverages [26]. However, while the inclusion of a diet rich in fruit and vegetables may have a preventive role in the pathogenesis of acute leukaemia, it is also reported that in the case of overt disease, therefore, in patients with acute leukaemia, a neutropenic diet, aimed at limitation of introducing bacteria into the gastrointestinal tract of the host through the limitation in particular of fresh fruit and vegetables, does not have a preventative role on the onset of infections, mortality or change in stool microbial flora [27].

Furthermore, apart from maternal diet, many studies have been conducted with children to evaluate the possible link of childhood leukaemia with dietary risk factors. At the Children’s Hospital, Pakistan Institute of Medical Sciences, Islamabad, Pakistan, in a case-control study carried out from January to December 2017, in which 2–12 year-old children were enrolled with recently diagnosed ALL or AML, including healthy children as a control group, the authors reported the intake of very high amounts of junk food and caffeinated drinks in the patients [28]. Considering that an unbalanced diet is associated with conditions such as obesity, one cannot fail to highlight the possible role of obesity as a main contributor to cancer risk, which can be prevented. Kinkaid JWR et al. reported that diet-induced obesity (DIO) hastens the progress of acute promyelocytic leukaemia (APL) in both male and female genetically predisposed mice [29].

Another important aspect to consider is the possible connection between breastfeeding and childhood leukaemia, for which an inverse relationship is reported that is explained by some biological mechanisms. Breastmilk has a prebiotic effect on the intestinal microbiota of the breastfed baby, in particular thanks to the secretory IgA antibodies it contains. Moreover, breastmilk contains lactoferrin, which plays a key role against microbes and a significant number of natural-killer cells, suggesting that formula-fed infants present a less mature immune system than breastfed children. Moreover, breastfed children reported a stomach pH level that is better in inducing the production of the protein-lipidα-lactalbumin (termed HAMLET), which is involved in apoptosis like death of tumour cells. Studies have reported that around 14–19% of all childhood leukaemia cases might be prevented if children are breastfed for at least 6 months [30].

## 3. Maternal Diet Quality and the Risk of Childhood Leukaemia: What Is the Possible Mechanism?

Gut microbiota is heavily modulated by diet: modulation during pregnancy is currently of great interest, considering the possible role it might play on maternal and neonatal health. Indeed, diet can alter the microbiota composition very quickly, in less than a week [31]. New evidence has reported that specific nutrients exert different actions on metabolic outcomes, depending on individual microbial patterns subject to specific individuals or conditions, suggesting the important role of a personalizing human nutrition treatment [32].

Throughout a healthy pregnancy, gut microbiota composition undergoes some alterations. In particular, decreased richness (intra-individual or α-diversity) and increased between-individual diversity (β-diversity) has been observed, with the presence in late pregnancy of microbial patterns that are comparable to those of non-pregnant women with metabolic syndrome. Moreover, during pregnancy an increase of *Proteobacteria*/*Actinobacteria*, a reduction of *Roseburia intestinalis* and *Faecalibacterium praunitzii* and α-diversity is typical of the microbiota composition [33]. The presence of dysbiosis induces metabolic abnormalities, for example abnormal gut permeability, which increases absorption of lipopolysaccharide (LPS) and abnormal SCFA production. There is also increased production of bacterial toxic substances (TMAO), leading to the activation of autoimmune and inflammatory pathways [31,32]. Therefore, alterations of the maternal microbiome would reflect on the neonatal gut microbiome. The neonatal gut microbiome primarily derives from the maternal gut, considering that vaginal microbiota and maternal skin only colonize the newborn baby in a transient way [34]. There are reports that intrapartum antibiotics, caesarean section, and formula feeding could disrupt microbiome establishment, inducing an increased risk of chronic inflammation and infectious exposures in a later stage, with the possible risk of ALL [34,35].

Maternal diet quality may play a key role in the possible risk of childhood leukaemia through various mechanisms, such as the impact of certain nutrients, such as folic acid, through epigenetic processes, DNA synthesis and repair, or mechanisms that impact children’s immune system development, before and after birth [36,37].

The immune response of the mother greatly impacts foetal immune development, and thus, incorrect maternal immune activation, which might be related to elevated levels of pro-inflammatory cytokines, can promote an augmented risk in the onset of many autoimmune diseases, neurodevelopmental disorders, and allergies, at a later stage [38,39]. Furthermore, an alteration in maternal cytokine production may induce foetal resorption [40]. Indeed, in mice models, the presence of increased levels of pro-inflammatory cytokines such as tumour necrosis factor (TNF)-α (a T helper (Th)1 cytokine) and interferon (INF)-γ and have been related to miscarriages [41].

Instead, health during pregnancy is related to an increased expression of humoral immunity, Th2 cytokines (e.g., IL-10, IL-13), and reduced cell-mediated immunity (Th1-type cytokines) in response to both foetal allo-antigens and environmental antigens [42].

The balance between Th17 cells and regulatory Tcells (Tregs) also plays a major role in maintaining maternal immune tolerance. Tregs have the ability to suppress both Th1and Th2 responses, while the role of Th17 cells is fundamental in autoimmune diseases and in protection against infections [43,44].

The foetal immune system is above all sensitive to alterations induced by the environment with particular factors acting on the maternal immune system, for instance, malnutrition. Development of the foetal immune system may be influenced by maternal malnutrition through diverse mechanisms which have only recently been hypothesized: (1) maternal malnutrition may induce elevated levels of maternal cortisol with a negative role on foetal immune system development; (2) low levels of micronutrients may alter processes such the normal proliferation of immune cells and organogenesis; and (3) reduced maternal nutrition might induce an alteration of the quantity and quality of immune factors that are passed on to the foetus via the placenta or subsequently through the mammary gland [45,46].

## 4. Microbiome and Acute Leukaemia: A Possible Relation?

The human microbiome, or the collective genetic information of microorganisms, both symbiotic and pathogenic, that populate the body, in particular the gut, have a major part to play in disease and health conditions, such as cancer, making up the “second genome” [47]. Many factors impact the make-up of gastrointestinal microbiota, for instance, heredity, environmental factors and lifestyle [48]. Several functions are mediated by gut microbiota, for example, the development of the immune system, digestion and absorption of food, involvement in the regulation of immunological processes and pathogen resistance, and synthesis of vitamins [49]. Moreover, intestinal microbiota homeostasis is involved in drug efficacy and side effects [50]. In literature, it has been reported that, in the population, the make-up of gut microbiota, which presents some parallels, can be modified by the start and progress of various diseases. The study of metagenomic samples from patients affected by different forms of cancer highlighted how microbiota might be used as bacterial markers for diagnosis in various diseases, for instance, *Fusobacterium nucleatum* and *Bacteroides fragilis* in colorectal cancer, and *Rikenellaceae*, *Akkermansia muciniphila* and *Bacteroides* in non-small cell lung cancer [51,52]. The development of several forms of cancer has been seen in association with microbial dysbiosis. Some studies reported a disequilibrium in the gut microbiota of ALL patients before treatment in comparison with healthy patients [2,3,53,54]. Dysbiosis of the gut microbiota induces the obliteration of the intestinal epithelial barrier, and some gut microbiota enter into local lymph nodes or the blood. These processes induce an inflammatory immune response that activates immune cells and involves metabolic pathways, contributing to the development of cancer [55,56,57]. One study reported a relevant difference in bacterial composition between a control group and a xenotransplant paediatric ALL mouse model in the faeces and small intestine, such as a relevant prevalence of bacteria with a conversion function of dietary flavonoids in the mouse model of ALL [58]. Rajagopala SV. et al. evaluated the composition of the GI microbiota in ALL adolescent and paediatric patients throughout chemotherapy. In particular, the authors compared stool samples before the start of chemotherapy with samples collected at different moments during chemotherapy. In both groups, the microbiota compositions were represented above all by *Faecalibacterium*, *Bacteroides* and *Prevotella*, and, while the authors reported a higher microbiota diversity in the control group than in the patient group. It was possible to identify the most frequent as *Coprococcus*, *Anaerostipes*, and *Ruminococcus2*, and *Roseburia* which were lower in abundance in the study group. These microbiota alterations are due to various factors, such as an indirect action of chemotherapy on the immune system and direct action of therapeutic compounds on the gut flora [59]. In literature, it has been reported that the assumption of bioflavonoids induces mixed lineage leukaemia (MLL) gene cleavage by targeting topoisomerase II, which might cause leukaemia in children [60,61]. Therefore, it can be understood how an abnormal gut microbiota make-up is closely related to the pathogenesis of leukaemia and how, in addition, alterations in the gut microbiota may be linked with genetic susceptibility to ALL, in ALL mouse models. Following this line, alterations in gut microbiota make-up were highlighted between mice genetically predisposed to leukaemia and healthy mice [62].

The relationship between cancer and microbes requires a thorough evaluation; indeed, it is possible that the various microbes localized at mucosal sites become part of the tumour microenvironment once barriers are breached, inducing cancer growth and then spreading using proinflammatory or immunosuppressive programs [52,63].

## 5. Microbiota and Drug Pharmacokinetics in Leukaemia

A significant factor related to drug effectiveness and possible side effects is intestinal microbiota homeostasis [48,49,50]. The complications and prognosis of acute leukaemia after hematopoietic stem cell transplantation or chemotherapy may be impacted by gut microbiota make-up; in particular, the use of probiotics, prebiotics and changes in diet may reduce the incidence of side effects related to the leukaemia treatment, improving efficacy and prognosis [49,60].

The link between chemotherapeutic drugs and gut microbiota is a matter of debate; for instance, chemotherapy drugs may act on the gut microbiota directly or induce injury to intestinal epithelial cells, causing an imbalance of the microbiota. But at the same time, dysbiosis of gut microbiota may have various effects on both the metabolism and absorption of chemotherapeutic drugs, with consequent increased toxicity and reduced efficacy [64,65]. In ALL patients, chemotherapy may induce some alterations in the gut microbiota make-up, with, in particular, a decrease of the diversity of gut microbiota and an increased presence of certain bacteria, for instance *Bacteroidetes*, while there is a reduction of other bacteria, for example *Streptococcaceae* and *Clostridiaceae*, as reported in a study conducted with 199 children affected by ALL who had undergone intensive induction [66,67]. In addition, similar research on the alteration of the composition of gut microbiota indicates an association with complications after chemotherapy, such as organ toxicity, neutropenia, infections and gastrointestinal dysfunction, influencing the prognosis of patients affected by leukaemia. High morbidity and mortality are related to febrile neutropenia [68,69].

In literature, an increased presence of opportunistic pathogens has also been described, for example, *Streptococcus*, *Staphylococcus*, *Lactococcus* and *Ralstonia*, and in the faeces of ALL mice when compared with control mice [58]. Moreover, injury to the intestinal epithelial barrier brought on by anti-tumour treatment and the abundance of some pathogens promotes the possible development of bacteraemia and resistant bacterial infections [60]. Some studies reported a close connection between alterations in gut microbiota and the level of intestinal epithelial loss and systemic inflammation and during chemotherapy [68]. Moreover, an increased level of Proteobacteria before treatment can be considered a predictive factor of febrile neutropenia: an increased presence of *Enterococcaceae* or *Streptococcaceae* was related to a higher risk of later infection in ALL patients, a reduction in alpha and beta diversities was related to an augmented risk of infection in the 6 months after therapy, and higher temporal variability of gut microbiota was related to an increased risk of infection at 3 months after induction in AML patients [70].

Regarding the concept of drug efficacy, the mutual association between gut microbiota and non-antibiotic drugs should be highlighted: the gut microbiome make-up may be influenced by drugs and vice versa [71,72]. Gut microbiome also plays a key role in influencing patient response to a drug, inducing an alteration in its structure and modifying its bioactivity, bioavailability, or toxicity [71]. In particular, gut microbiota may influence patient response to chemotherapeutic drugs during ALL treatment. For example, one of the strongest anticancer drugs, methotrexate (MTX), very frequently used for the treatment of leukaemia for its ability to block folate metabolism, can induce atrophy of jejunal villi, an increase of goblet cells, a collapse of the muscularis mucosa, an increase in inflammatory processes such as a dysregulation of macrophages (with a rise in M1 phenotype and a reduction in M2 phenotype) in lymph nodes and spleen. In addition, MTX may cause a change in the gut microbiota make-up in mice, above all a significant reduction in *Bacteroidales*. *Bacteroides fragilis* may induce an improvement in the polarization disequilibrium of macrophages and inflammatory response induced by MTX [72].

Some studies reported that non-high dose Cyclophosphamide (CTX), another broad-spectrum anti-tumour drug, in mice can induce alterations in the mouse intestine such as shortening of villi, discontinuity of the epithelial barrier, focal accumulation of monocytes in the lamina propria, interstitial oedema, and a rise in Paneth cells and goblet cells causing an increase in intestinal permeability, facilitation of intestinal bacterial translocation, leading thus to bloodstream infection (BSI). In particular, two bacterial species, *Barnesiella intestinihominis* and *Enterococcus hirae*, are reported to be involved in CTX therapy [73,74]. Viaud et al. (2013) reported an alteration of the microbiota make-up in the small intestine and a substantial translocation of commensal bacteria in the majority of mice which were administered CTX, with the detection of some Gram-positive bacteria species (for example, *Lactobacillus murinus*, *Lactobacillus johnsonii* and *Enterococcus hirae*) in lymphoid organs, for instance, spleens and mesenteric lymph nodes. The inhibition of tumour growth mediated by CTX can be decreased by antibiotic treatment, whilst adoptive transfer of pathogenic Th17 (pTh17) cells may eradicate this harmful effect of antibiotics, highlighting how gut microbiota might have a key role contributing to CTX-mediated anticancer immune responses through pTh17 cells, and Gram-positive bacteria may have a key role in contributing to anticancer immunological responses by stimulating Th17 cells [73].

A study that evaluated 271 oral drugs and 76 diverse human gut bacteria reported that the metabolism of various drugs, such as corticosteroids, is chemically modified by microbiota [75]. The use of gene sequencing in combination with mass spectrometry can recognise drug metabolic activities of human gut bacteria and microbial gene products that metabolize drugs. In this way, microbiota may be envisaged by analysing their genomic contents. In a multicentre retrospective study, the authors reported that antibiotic exposure of patients receiving a chimeric type of antigen receptor (CAR) T cell therapy, one of the main immunotherapies for ALL, was linked with worse overall survival (OS). An analysis of stool samples from 48 patients with lymphoma or leukaemia highlighted that patients undergoing CAR-T cell therapy with a higher efficacy of this therapy have an unusually low alpha diversity and an elevated presence of *Bacteroides*, *Ruminococcus* and *Faecalibacterium*. Moreover, the various microbiota data at diverse treatment times (for instance, subsequent to CAR-T cell therapy and during follow-up) might help clinicians in better defining the role of gut microbiota in CAR-T cell therapy [76].

Therefore, a personalized analysis of the individual differences of microbiota is necessary to understand the possible disparities in drug responses and drug metabolism, which can be modulated through the adjustment of exogenous microorganisms. However, further studies are needed to evaluate specific mechanisms of the interaction of bacterial metabolites and bacterial taxa in the immune system, so as to define and advance patient outcomes after specific treatment.

## 6. Vitamin D and Acute Leukaemia

Bone metabolism and calcium homeostasis are the principal biological activities of vitamin D. Nonetheless, considering that the vitamin D receptor (VDR), a member of the family of nuclear receptors, is present in various diverse cells and tissues of the human body, such as dendritic cells (DCs), it is important to remember that this vitamin performs many actions that can be classified as “non-classical actions”. In particular, vitamin D has a major role in regulating immune cell function and hematopoietic cell differentiation and proliferation. Considering these effects, vitamin D might represent a therapeutic strategy hematologic malignancy treatment [77]. Moreover, as reported by Murdaca G. et al., reduced values of vitamin D influence the microbiome, inducing an alteration of the integrity of the gut epithelial barrier and the microbiome composition. Both dysbiosis and vitamin D deficiency are related to chronic inflammation and an augmented risk of various diseases, for example, cancer. Changes in vitamin D/VDR signalling with microbiome dysbiosis have been reported to be related to both intestinal inflammatory processes and extra-intestinal disorders [78]. In particular, the action mediated by vitamin D is related to its capacity to act on cell function in the gut by binding to its intracellular VDR, inducing the transcription of many genes. In the lumen, vitamin D has a key part in the maintenance of the correct level of antimicrobial peptides in the mucus and in maintaining epithelial integrity acting on the intercellular junctions. When bacteria infiltrate the epithelial layer and arrive at the interstitium, inflammation activates Th1/Th17 cells. In these conditions, the activation of vitamin D/VDR signalling allows the clearance of the bacteria in the affected cells with the suppression of Th1/Th17 cells and activation of Treg cells [79].

Many studies reported the use of vitamin D and its analogues in myeloid neoplasms, above all in AML and myelodysplastic syndrome (MDS) treatment. Some preclinical experiences with HL-60 and other leukemic lines, for example U-937 and THP-1, reported that vitamin D induces differentiation and apoptosis of blasts. In particular, Tanaka H. et al. showed that the vitamin D analogue induces an improvement in survival in leukemic mice [80]. Muto A. et al. highlighted the efficacy of calcitriol in inhibiting the cell cycle and inducing leukaemia cell differentiation through VDR [81]. Moreover, in literature, it has been reported that vitamin D induces growth inhibition and cell differentiation in leukaemic cell lines or AML blasts [82].

Numerous studies have tried to analyse the precise mechanism of action regarding the activation of VDR, theorizing the involvement of various pathways involving the MAPK pathway, P13 kinase, and possibly the upregulation of factors like p53. In a retrospective study, a correlation between VDR expression and prognosis was reported. In particular, higher VDR expression has been related to increased survival. Moreover, the authors reported a correlation between patient prognosis and expression of VDR-targeted genes, such as the finding that patients with higher cyclic adenosine monophosphate (cAMP) expression are associated with higher event-free survival (EFS), unlike patients with reduced cAMP expression levels [83].

Motomura et al., analysing 30 patients with myelodysplastic syndromes (MDS) undergoing treatment with 25(OH)D3 as opposed to supportive treatment, reported that just one of the fifteen vitamin D group patients reported progression to AML as against seven in the control group [84]. In some studies, the combination of vitamin D with other cytotoxic agents was evaluated. Siitonen et al., treating 19 MDSs patients using a combination of 1,25(OH)2D3 (1 mg/day),13-cis retinoic acid, and valproic acid, reported that three patients had a haematological response. However, due to 13-cis retinoic acid and valproic acid, some intolerance was highlighted in eight patients, [85].

Therefore, considering the capacity of vitamin D and its analogues to drive the differentiation of immature myeloid hematopoietic cells into mature monocytic cells, in literature, it has been reported that only in very few patients is there either a persistent or transient progress in blood counts using vitamin D and analogue therapy. However, some additional studies would be needed to evaluate this approach in the formal medical treatments for MDS and AML [86,87,88].

## 7. Possible Therapeutic Strategies for the Recovery of Microbiota in Leukaemia Patients

Probiotics, active microorganisms that modulate immunity or block intestinal inflammation, decreasing intestinal dysbiosis, have been amply adopted to manage intestinal alterations and adverse results induced by various treatments or conditions in patients affected by diseases such as leukaemia. In a randomized controlled trial conducted on 60 children affected by ALL treated with chemotherapy, a relevant decrease in many gastrointestinal side effects, such as nausea, vomiting, flatulence and abdominal distension/pain, was reported in some patients treated with oral probiotics (*Lactobacillus rhamnosus*), taken daily, and chemotherapy [89]. Faecal microbiota transplantation (FMT), consisting of the transfer of stool through medically or self-dispensed enemas, oral capsules, via nasogastric tube, or instilled into the duodenum by upper endoscopy or the right colon by colonoscopy or cecostomy, from a healthy donor into the colon of the recipient, has the aim to restore the microbiota composition according to different patients and conditions [90,91]. In a clinical trial conducted on four ALL patients undergoing allo-HSCT with refractory diarrhoea due to refractory intestinal infection or intestinal graft-versus-host disease, a complete remission induced by FMT in three patients was reported, while one was stable [92]. In literature, it was also reported that the use of prebiotics, such as *Lactobacillus rhamnosus*, during chemotherapy may help patients restore the altered microbiota after treatment, reducing the therapeutic side effects. A study reported how also a melatonin supplement can induce an augmented diversity of gut microbiota, regulating the make-up, that increases the presence of *Lactobacillus* in mice, suggesting that melatonin-enriched food can play a major part in remodulation of gut microbiota after chemotherapy in leukaemia patients [93].

Therefore, an appropriate diet and the assumption of prebiotics can be considered as alternative strategies of support for the management of altered microbiota in leukaemia patients.

However, it is important to highlight the concept of safety of probiotic products that is related to factors such as infectivity, pathogenicity, excessive immune stimulation in susceptible patients and virulence factors (e.g., toxicity, metabolic activity). Literature reports on side effects triggered by the use of probiotic bacteria that can be involved in systemic infections, in disturbing metabolism, in the stimulation of the immune system, and participating in horizontal gene transfer [94].

Moreover, it is important to highlight that amongst the various mechanisms through whereby an altered microbiota might induce the onset and progression of neoplastic diseases, there is the role of dysbiosis that can provoke lipid metabolism-related microRNA (miRNA) expression change [95].

Therefore, considering the existence of a bidirectional relationship between miRNAs, a novel group of small endogenous non-coding miRNA molecules that are involved in gene expression through base complementarity between the seed region of the miRNA and the 3′-untranslated region (UTR) of the target mRNA, and microbiota, it is more and more evident how miRNA modulation might become a significant therapeutic opportunity for the treatment of many diseases [96,97].

## 8. Conclusions

Our analysis suggests the key role of gut microbiota in the modulation of the efficacy of leukaemia treatment, and, more importantly, in the development of some cancers, such as acute leukaemia. Although an increasing number of studies are dealing with the possible role of the microbiota as a targeted therapy in many neoplastic diseases, there is scanty data on the relevance of microbiota and diet changes in leukaemia. In particular, as reported above and in Table 1, many studies investigated the role of diet on the development and prevention of acute leukaemia and the possible role of a diet high in vegetables and fruit in leukaemia development due its significant effects on altering microbiota and immunological pathways [13,19,26,27,28,29,61,98,99,100,101,102,103,104,105,106].

However, while prevention represents one of the weapons on which we are increasingly trying to act to limit the development of neoplasms, particularly during adulthood, a different discussion could take place for childhood cancer, when talking about both leukaemia and solid tumours. Indeed, in these cases, the identification of causal factors is extremely difficult, given that neoplastic conditions are biologically diverse and rare in prevalence [107]. Furthermore, for most childhood cancers, there is the possibility of underlying stochastic developmental errors compounded by inherited susceptibility [2,53,108].

An exception to this pessimistic consideration is childhood ALL, for which a risk factor might be the lack of exposure to microbes at a very early age. In particular, protective factors against the appearance of ALL include daycare attendance and protracted breastfeeding, while among increased risk factors, there is caesarean section birth [34,107,108]. Evidence shows that the gut microbiome make-up at birth and during the early years of life have relevant, long-lasting effects on the immune system as a result of many mechanisms, for instance, direct microbial binding to toll receptors on innate immune cells, the role of metabolic products of bacteria, and the consequent activation of regulatory T cells [109,110,111].

Moreover, in this paper, considering that in literature it has been reported that vitamin D induces cell differentiation and growth inhibition on AML blasts or leukemic cell lines, we investigated the role of vitamin D use and its analogues in particular in myeloid neoplasms inducing, or not, a haematological response.

Much research on the mother’s diet and leukaemia risk in childhood hasalso analysed the function of diet quality in influencing childhood leukaemia risk. Indeed, as already reported, the risk of leukaemia in childhood may be influenced by maternal nutrition, both through taking maternal folic acid and maternal consumption of specific food groups during pregnancy, due to its role in foetal development, such as the synthesis and repair of DNA, inducing epigenetic mechanisms.

An important aspect to highlight is that most of the cited studies are performed on cell lines or mice, with a lack of studies on large groups of patients, thus supporting the need for further studies and analysis to ascertain evidence of the link between diet, microbiota and leukaemia and how to act with specific dietary interventions and therapeutic strategies. In particular, further studies are needed to confirm whether or not that in patients developing ALL the microbiome is lacking or deficient in diversity, considering, undoubtedly, possible potential confounding effects, such as the disease process itself or prior antibiotic use.

From our analysis we see that the impact of the microbiome and changes in diet through certain immunological pathways might be tumour-specific, and this may represent a future approach of precision medicine in the management and prevention of leukaemia.

Therefore, if improvement in the prognosis of leukaemia patients is a goal of the management of this disease, we can understand how restoring the altered microbiota can be considered a key tool for the patient following an approach of precision and personalized medicine.

## Figures and Tables

**Table 1:** Main articles regarding the role of diet on the development and prevention of acute leukaemia.

Authors	Paper	Main Results
[13]	High salt diet does not impact the development of acute myeloid leukaemia in mice.	A high-salt diet induces a significant change in microbiota composition, TH17 responses, and NK cells, not inducing a significant role in tumour development.
[19]	Maternal diet quality before pregnancy and risk of childhood leukaemia.	Higher maternal diet quality score was associated with a reduced risk of acute myeloid leukaemia.
[26]	Role of Maternal Diet in the Risk of Childhood Acute Leukemia: A Systematic Review and Meta-Analysis.	Maternal consumption of fruit was inversely associated with acute lymphoblastic leukaemia, while maternal coffee intake was associated with an increased risk of acute lymphoblastic leukaemia.
[27]	Neutropenic versus regular diet for acute leukaemia induction chemotherapy: randomised controlled trial.	A neutropenic diet did not play a preventive role in infections, reducing mortality or changing stool microbiota composition in patients with acute leukaemia.
[28]	Evaluation of diet as a risk factor in the development of childhood leukaemia: a case-control study.	Higher consumption of caffeinated drinks and junk food was reported in children of either gender aged 2–12 years with acute lymphocytic or acute myelocytic leukaemia.
[29]	Obesity accelerates acute promyelocytic leukaemia in mice and reduces sex differences in latency and penetrance.	In vivo, obesity and obesogenic diet play a key role in promoting the development of acute promyelocytic leukaemia.
[61]	Dietary bioflavonoids induce cleavage in the MLL gene and may contribute to infant leukaemia.	Maternal ingestion of bioflavonoids may induce MLL breaks and may induce translocations in utero, causing infant and childhood leukaemia.
[98]	High-fat diet intensifies MLL-AF9-induced acute myeloid leukaemia through activation of the FLT3 signalling in mouse primitive hematopoietic cells.	Using an MLL-AF9 knock-in mouse model, consumption of a high-fat diet accelerates the risk of developing acute myeloid leukaemia, with increased clusterization of FLT3 within lipid rafts on cell surface of primitive hematopoietic cells and consequent overactivation of JAK/STAT pathway.
[99]	Fatty acid-binding protein FABP4 mechanistically links obesity with aggressive AML by enhancing aberrant DNA methylation in AML cells.	Leukaemia burden was much higher in high-fat diet-induced obese mice, in which increased levels of FABP4 and interleukin (IL)-6 in sera were described.
[100]	Dietary intake of vegetables, fruits, and meats/beans as potential risk factors of acute myeloid leukaemia: a Texas case-control study.	A decreased risk of acute myeloid leukaemia was reported among patients who consumed higher quantities of dark green vegetables, seafood, and nuts/seeds. The risk was instead significantly increased among greatest consumers of red meat.
[101]	Dietary factors and the risk for acute infant leukaemia: evaluating the effects of cocoa-derived flavanols on DNA topoisomerase activity.	In vitro data reported that cocoa-derived flavanols have limited effects on topo II activity and cellular proliferation in cancer cell lines, with a leukemogenic potential at physiological concentrations.
[102]	Diet, lifestyle, and acute myeloid leukaemia in the NIH-AARP cohort.	Increased risk of acute myeloid leukaemia was related to a higher meat intake; patients who did not drink coffee appeared to have a higher risk of acute myeloid leukaemia than those who drank various quantities of coffee; the intake of fruit and vegetables was not related to acute myeloid leukaemia.
[103]	Maternal diet and infant leukaemia: a role for DNA topoisomerase II inhibitors?	There was no positive correlation with increasing maternal assumption of DNA topo 2 inhibitor-containing foods (specific fruits and vegetables, and in soy, coffee, wine, tea and cocoa) neither for the overall group nor for infants in the acute lymphoblastic leukaemia stratum.
[104]	Intake of selected food groups and beverages and adult acute myeloid leukaemia.	The risk of AML was negatively related to the assumption of milk and tea among women and positively associated with the intake of beer, wine, and beef.
[105]	Diet and risk of leukaemia in the Iowa Women’s Health Study.	Risk of adult leukaemia may be decreased by increased vegetable consumption.
[106]	Dietary and other environmental risk factors in acute leukaemias: a case-control study of 119 patients.	Low consumption of raw vegetables, frequent drinking of milk, frequent consumption of poultry, and drinking of soft water were related to an increased risk of acute leukaemia.

## Data Availability

No new data were created or analyzed in this study. Data sharing is not applicable to this article.
